# The effect of leukoreduction and prolonged storage on coagulation in cold‐stored whole blood: An in vitro study

**DOI:** 10.1111/vox.70075

**Published:** 2025-08-05

**Authors:** Sanna Susila, Teemu Silver, Tuukka Helin, Timo Jama, Jouni Lauronen, Lotta Joutsi‐Korhonen, Minna Ilmakunnas

**Affiliations:** ^1^ Finnish Red Cross Blood Service Vantaa Finland; ^2^ Emergency Medical Service and Emergency Department Päijät‐Häme Wellbeing Services County Lahti Finland; ^3^ Department of Anesthesiology and Intensive Care Medicine Helsinki University Hospital and University of Helsinki Helsinki Finland; ^4^ Department of Clinical Chemistry, HUS Diagnostic Center Helsinki University Hospital and University of Helsinki Helsinki Finland

**Keywords:** blood storage, cold‐stored whole blood, haemostasis, leukoreduction, whole blood

## Abstract

**Background and Objectives:**

Cold‐stored whole blood (CSWB) for haemodynamically unstable bleeding patients is especially convenient when platelets are otherwise unavailable and simple logistics are favoured, as in prehospital situations. Storage times exceeding the currently common 21 days would be beneficial in situations where normal blood processing and distribution is disturbed. To better understand extended‐storage CSWB haemostatic properties, we studied leukoreduced CSWB (LR‐CSWB) and non‐leukoreduced CSWB (non‐LR‐CSWB) for 5 weeks of cold storage.

**Materials and Methods:**

Non‐LR‐CSWB was donated by seven donors and refrigerated for 5 weeks. Eight units of 20‐day‐old LR‐CSWB returned from clinical rotation unused were stored for an additional 2 weeks. Previously published results were used for early‐storage LR‐CSWB data. Blood counts, coagulation assays, multiple electrode aggregometry, thrombin generation, rotational thromboelastometry and sonorheometry were analysed.

**Results:**

Regardless of leukoreduction, markedly reduced platelet aggregation and increasing FXIII levels as a sign of platelet activation were seen during storage. Coagulation factors generally decreased and clotting times increased during storage, but endogenous thrombin potential remained normal at 5 weeks in both groups. Viscoelastic assays displayed conflicting results later during storage, with extremely low clot stiffness in sonorheometry concomitantly with apparently adequate clotting in thromboelastometry.

**Conclusion:**

Most haemostatic changes in LR‐CSWB and non‐LR‐CSWB occur within the first 2 weeks of storage. Little difference remains between LR‐CSWB and non‐LR‐CSWB aged 5 weeks. Differences in viscoelastic properties suggest that clot strength may be weaker than thought already in 14‐day‐old CSWB regardless of leukoreduction.

**Highlights**

- Most haemostatic changes in cold‐stored whole blood (CSWB) occur during the first 14 storage days.
- Leukoreduction does not significantly alter haemostatic capacity in CSWB after prolonged storage.
- Clot structure may be weaker than previously thought already in 14‐day‐old CSWB.

## INTRODUCTION

Cold‐stored whole blood (CSWB) is increasingly used as the first‐line blood product in haemorrhagic shock, especially in civilian prehospital care and remote areas, where the requirements of simple storage facilities, ease of use and the availability of platelets are all in favour of CSWB [1].

Currently, CSWB can be stocked in citrate‐phosphate‐dextrose (CPD) for 21 days or in citrate‐phosphate‐dextrose‐adenine (CPDA‐1) for 35 days. Blood collected in CPD can be leukoreduced with a built‐in filter available in commercial collection sets, whereas leukoreduction is currently unavailable for CPDA‐1 collection sets. In exceptional circumstances where the normal blood processing and distribution chain is insufficient or compromised, it may be desirable to produce CSWB with as long a storage time as possible. As leukoreduction decreases the probability of adverse transfusion‐related reactions, including non‐haemolytic febrile reactions and cytomegalovirus infections [2], the civilian use of leukoreduced CSWB (LR‐CSWB) is reasonable. Non‐leukoreduced CSWB (non‐LR‐CSWB) may be used when blood processing is not feasible.

Previous in vitro assessment of CSWB has mostly focused on WB stored in CPD for the recommended storage time of 21 days [3, 4, 5, 6, 7, 8, 9]. Extended storage for up to 35 days has been evaluated in CPDA‐1 and CPD, leukoreduced and non‐leukoreduced [10, 11, 12, 13, 14]. However, knowledge on the haemostatic properties of LR‐CSWB after extended storage is still insufficient as platelet contribution to clot formation is mostly assessed by two similar viscoelastic methods: rotational thromboelastometry (ROTEM) and thromboelastography (TEG). We designed a study comparing the haemostatic properties of LR‐CSWB stored in CPD and non‐LR‐CSWB stored in CPDA‐1 for 35 days, including sonorheometry, platelet aggregometry, von Willebrand factor (VWF) and coagulation factor assays. We hypothesized that, despite prolonging storage of LR‐CSWB from the currently recommended 21 to 35 days, the haemostatic function of LR‐CSWB is sufficient and comparable to non‐LR‐CSWB.

## MATERIALS AND METHODS

Non‐LR‐CSWB was collected from seven adult male O RhD positive blood donors recruited for research purposes. Use of non‐steroidal anti‐inflammatory drugs and herbal medications was prohibited 2 weeks prior to donation. Standard Finnish Red Cross Blood Service (FRCBS) donation criteria and donation practices were used. Haemoglobin was checked before donation, with a median of 152 (range 142–161) g/L. On day 0 (d0), WB was collected into Terumo PB‐1CD456M0Y bags (Terumo Penpol Pvt. Ltd., Thiruvananthapuram, India) containing 63 mL of CPDA‐1. Blood was cooled and stored in +5°C within 2 h after donation, without leukoreduction.

LR‐CSWB units (*n* = 8) were 20‐day‐old low‐titre group O whole blood (LTOWB) returned unused from clinical rotation to FRCBS, prior to official unit expiry. These units were used for d21, d28 and d35 analyses. LR‐CSWB units were stored at +5°C. For d1 and d14 analyses, we used previously published data [15, 16]. All leukoreduced units were collected with a Terumo Imuflex® WB‐SP collection set (Terumo Europe N.V., Leuven, Belgium) containing 63 mL of CPD. These units were stored at room temperature prior to processing, leukoreduced 18–24 h after donation with a built‐in platelet‐sparing filter and refrigerated at +5°C only after leukoreduction.

The study was approved by the HUS Helsinki University Hospital regional medical ethics committee (HUS/5671/2023) and HUS Diagnostic Center (HUS/31/2024). An informed consent was obtained from all non‐LR‐CSWB donors.

### Blood samples

Samples from blood bags on d1, d14, d21, d28 and d35 after donation were collected in a sterile manner into non‐anticoagulated tubes (BD Vacutainer No Additive Z, Becton Dickinson Finland, Vantaa, Finland) and processed within 3 h of collection. On d1 in the non‐LR‐CSWB, the samples were drawn prior to cooling.

Control samples to determine baseline blood counts and thrombin generation were drawn from the pre‐donation bag to appropriate tubes (BD Vacutainer K2 EDTA and BD Vacutainer 3.2% Citrate, Becton Dickinson Finland).

Citrated blood for coagulation assays was centrifuged at 2500 g for 10 min. Coagulation tests were analysed immediately, except for factor (F) X, FXIII and protein C (PC), which were stored at −20°C until analysis. For thrombin generation assay, plasma was additionally centrifuged at 2500 g for 10 min before freezing at −80°C until analysis.

### Laboratory analyses

Blood counts were analysed with Sysmex XN‐9000 analyser (Sysmex Corporation, Kobe, Japan). Plasma haptoglobin and haemolysis index (H‐index [17, 18]) were analysed with Atellica CH 930 Analyzer (Siemens Healthineers, Erlangen, Germany). Activated partial thromboplastin time (APTT), antithrombin (AT3), fibrinogen, FV, FVIII, FX, FXIII, PC, prothrombin time (PT) and VWF glycoprotein Ib activity (VWF:Act) and antigen (VWF:Ag) were analysed in HUSLAB accredited hospital laboratory using Siemens Sysmex CS5100S platform (Siemens Healthineers, Erlangen, Germany). Due to updated laboratory equipment between LR‐CSWB d1 and d14 versus later samples, APTT results for LR‐CSWB d1 and d14 samples were corrected with the laboratory‐specific factor of −8% (written communication, Tuukka Helin 16.1.2025). PT, reported in percentage, decreases as clotting time (CT, in seconds) increases.

Multiple electrode aggregometry (MEA) was performed in duplicate with Multiplate analyser (Roche, Basel, Switzerland). Responses to adenosine diphosphate (ADPtest) at 6.5 μM and thrombin receptor‐associated peptide‐6 (TRAPtest) at 32 μM were assessed. Data are presented as area under curve (AUC).

Viscoelastic tests were done with ROTEM sigma analyser (Werfen, Barcelona, Spain) and sonorheometry Quantra Hemostasis System (HemoSonics, LLC, USA). ROTEM cassettes for extrinsic pathway (EXTEM; activator: tissue factor), intrinsic pathway (INTEM; activator: ellagic acid) and fibrin formation (FIBTEM) were used and CT, amplitude at 5 min (A5), maximum clot firmness (MCF) and maximum lysis (ML) were reported. EXTEM‐FIBTEM MCF, a surrogate measure for platelet contribution to clot formation, was calculated by subtracting FIBTEM MCF from EXTEM MCF. Quantra QStat cartridges were used and CT (activator: kaolin), clot stiffness (CS; activator: tissue factor), platelet contribution to CS (PCS), fibrinogen contribution to CS (FCS) and clot stability to lysis (CSL) were reported. The detection limit for CS, PCS and FCS was 2.0 hPa, and results <2.0 hPa were entered in data as 1.9 hPa.

Thrombin generation in platelet‐poor plasma was assessed with calibrated automated thrombogram (CAT, Diagnostica Stago, Asnieres, France). To measure maximal thrombin generation, 5 pM tissue factor was used, without thrombomodulin addition. Lag time, time to peak, thrombin peak and endogenous thrombin potential (ETP) were reported.

### Statistical analysis

Statistical analyses were done using IBM SPSS Statistics, version 28. Due to small sample size, non‐parametric tests were used. Differences between groups were tested with the Mann–Whitney *U* test. Time‐dependent changes during LR‐CSWB and non‐LR‐CSWB storage were tested with Friedman's test, and if significant, compared pairwise with Wilcoxon's test with Holm's correction. LR‐CSWB was tested with Friedman's test for LTOWB only, that is, excluding d1 and d14 data from previous studies. To compare LR‐CSWB d1 results with d21, d28 and d35 results, the Mann–Whitney *U* test with Bonferroni correction was used. A *p*‐value of <0.05 was considered statistically significant.

## RESULTS

### Cell counts and haemolysis

**FIGURE 1:**
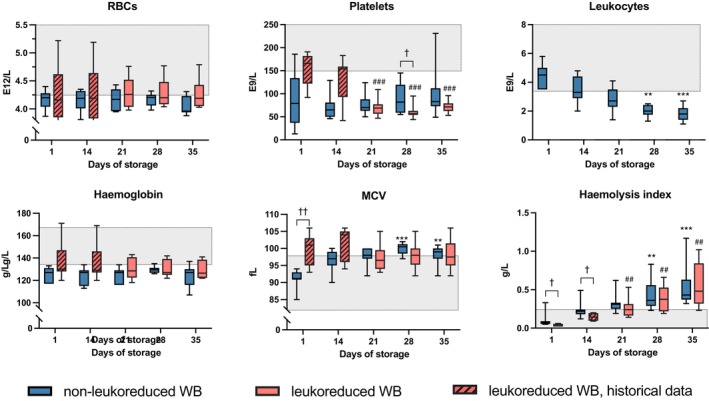
Haemostatic parameters in cold‐stored whole blood (CSWB). Grey area indicates laboratory reference range. Changes during storage in non‐leukoreduced CSWB (non‐LR‐CSWB) between day 1 and subsequent days are indicated by *p < 0.05, **p < 0.005, ***p < 0.001. Changes during storage in leukoreduced CSWB (LR‐CSWB) between day 1 and days 21, 28 and 35 are indicated by ^#^ *p* < 0.05, ^##^ *p* < 0.005, ^###^ *p* < 0.001. Differences between LR‐CSWB and non‐LR‐CSWB are indicated by ^†^ *p* < 0.05, ^††^ *p* < 0.005. There were no detectable leukocytes in LR‐CSWB. Haemolysis index interpretation: <0.25 g/L normal serum, 0.25–0.50 g/L insignificant haemolysis, 0.5–3.0 g/L mild haemolysis, 3.0–5.0 g/L moderate haemolysis, >5.0 g/L gross haemolysis. RBCs, red blood cells; MCV, mean cell volume.

In non‐LR‐CSWB, red blood cell (RBC) counts remained stable during storage. Initial and end‐of‐storage haemoglobin content remained similar. Haematocrit (median 38% [interquartile range, IQR 37%–39%] on d1, 40% [38%–42%] on d35) and mean cell volume (MCV) (*p* < 0.001; Figure 1) increased significantly. H‐index (*p* < 0.001) increased, but the median was 0.43 on d35, indicating insignificant haemolysis. Haptoglobin decreased slightly from 0.59 to 0.56 g/L (*p* = 0.005), remaining within reference range. Platelet counts remained stable (Figure 1). The observed d1 platelet count range (13–186 E9/L) was remarkably wide with subsequent increases in some samples. Leukocyte counts decreased (*p* < 0.001; Figure 1).

In LR‐CSWB, RBC counts and haemoglobin remained stable during storage. Haematocrit (41% on d1 [previously published data], 41% on d35) and MCV (Figure 1) remained stable. H‐index increased (*p* = 0.004; Figure 1), reaching a median of 0.48 on d35, indicating insignificant haemolysis. Haptoglobin was stable (0.66 g/L on d1, 0.45 g/L on d35). Platelet counts decreased (*p* < 0.001; Figure 1). There were no detectable post‐leukoreduction leukocytes (detection limit 0.03 E9/L).

Comparing non‐LR‐CSWB and LR‐CSWB, cell counts and haemolysis markers were generally similar in both groups (Figure 1).

### Clotting factors

**FIGURE 2:**
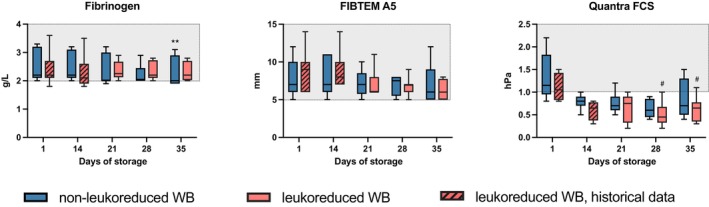
Fibrinogen analyses in cold‐stored whole blood (CSWB). Grey area indicates laboratory reference range. Changes during storage in non‐leukoreduced CSWB between day 1 and subsequent days are indicated by **p < 0.005. Changes during storage in leukoreduced CSWB between day 1 and days 21, 28 and 35 are indicated by ^#^ *p* < 0.05. A5 amplitude at 5 min, FCS, fibrinogen contribution to clot stiffness.

**FIGURE 3:**
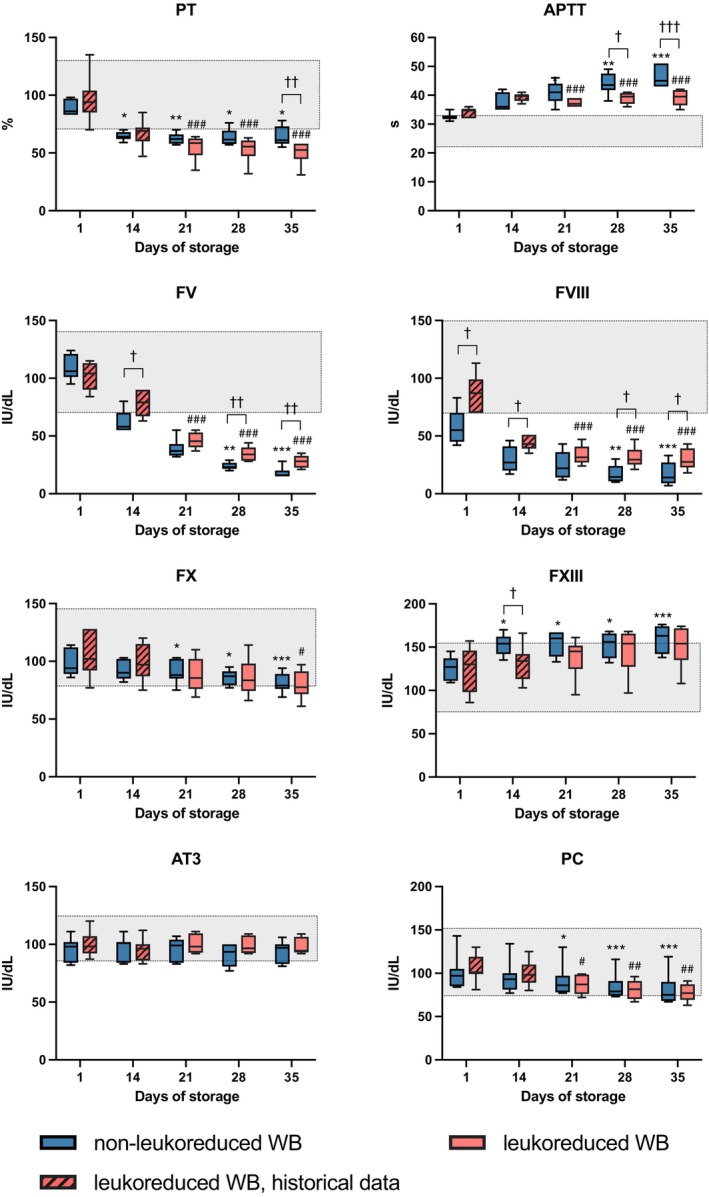
Coagulation assays in cold‐stored whole blood (CSWB). Grey area indicates laboratory reference range. Changes during storage in non‐leukoreduced CSWB (non‐LR‐CSWB) between day 1 and subsequent days are indicated by *p < 0.05, **p < 0.005, ***p < 0.001. Changes during storage in leukoreduced CSWB (LR‐CSWB) between day 1 and days 21, 28 and 35 are indicated by ^#^ *p* < 0.05, ^##^ *p* < 0.005, ^###^ *p* < 0.001. Differences between LR‐CSWB and non‐LR‐CSWB are indicated by ^†^ *p* < 0.05, ^††^ *p* < 0.005, ^†††^ *p* < 0.001. APTT, activated partial thromboplastin time; AT3, antithrombin; F, factor; PC, protein C; PT, prothrombin time.

In non‐LR‐CSWB, a statistically significant (*p* = 0.001) but clinically likely irrelevant change was observed in fibrinogen level during storage (Figure 2). A notable decrease in FV and FVIII and a lesser decrease in FX levels were observed (*p* < 0.001; Figure 3). These changes were reflected in prolonged APTT (*p* < 0.001) and decreasing PT (*p* = 0.011). FXIII increased (*p* = 0.002; Figure 3), mainly during early storage. Statistically significant but clinically likely irrelevant changes were seen in AT3 (*p* = 0.005) and PC levels (*p* < 0.001).

In LR‐CSWB, fibrinogen levels remained stable during storage. FV and FVIII levels decreased notably (*p* < 0.001). A lesser decrease in FX levels was observed. APTT prolonged and PT decreased (*p* < 0.001). FXIII increased during storage (*p* = 0.01). There was no change in AT3, but PC levels decreased (*p* = 0.005).

Comparing non‐LR‐CSWB and LR‐CSWB (Figure 3), FV and FVIII levels decreased more in non‐LR‐CSWB. Thus, APTT was longer in non‐LR‐CSWB on d28 (*p* = 0.013) and d35 (*p* < 0.001). While no difference in FX was observed, PT was higher in non‐LR‐CSWB than in LR‐CSWB on d35 (*p* = 0.006).

### VWF and platelet function

**FIGURE 4:**
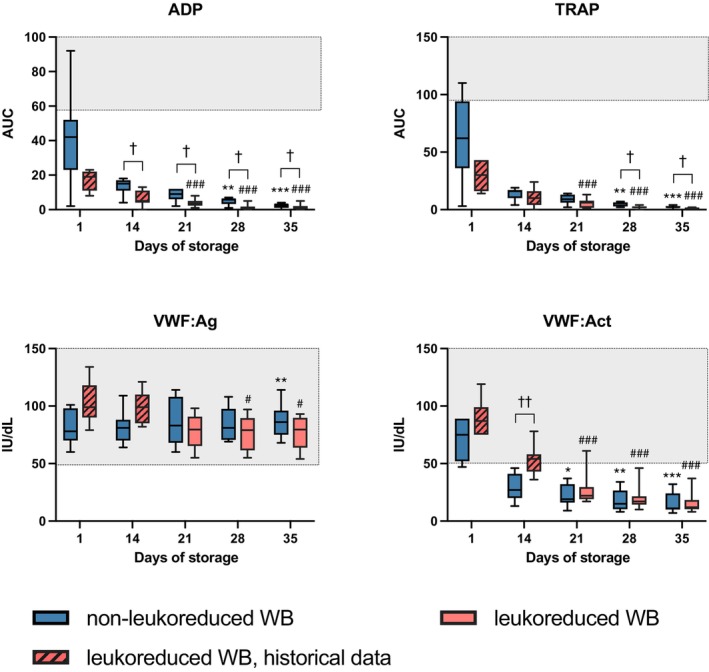
Platelet function in cold‐stored whole blood (CSWB). Grey area indicates laboratory reference range. Changes during storage in non‐leukoreduced CSWB (non‐LR‐CSWB) between day 1 and subsequent days are indicated by *p < 0.05, **p < 0.005, ***p < 0.001. Changes during storage in LR‐CSWB between day 1 and days 21, 28 and 35 are indicated by ^#^ *p* < 0.05, ^###^ *p* < 0.001. Differences between leukoreduced and non‐leukoreduced samples are indicated by ^†^ *p* < 0.05, ^††^ *p* < 0.005. Act, activity; ADP, adenosine diphosphate; Ag, antigen; AUC, area under curve; TRAP, thrombin receptor associated peptide‐6; VWF, von Willebrand factor.

In non‐LR‐CSWB, responses to ADP and TRAP in MEA declined rapidly during storage (*p* < 0.001). A slight late increase in VWF:Ag (*p* = 0.034; Figure 4) and a gradual decrease in VWF:Act was observed (*p* < 0.001).

In LR‐CSWB aged 21 days or more, responses to ADP and TRAP in MEA declined compared to d1 (*p* < 0.001). VWF:Ag remained relatively stable. VWF:Act decreased gradually (*p* < 0.001).

Comparing non‐LR‐CSWB and LR‐CSWB, the ADP and TRAP responses were lower throughout storage in LR‐CSWB, although responses were substantially decreased already on d14 in both groups (Figure 4). No major differences in VWF assays were observed.

### Thrombin generation

Compared with baseline values determined from d0 donor samples, time to peak was prolonged in non‐LR‐CSWB on d1 (*p* = 0.008). No statistically significant difference in lag time, thrombin peak or ETP was observed.

**FIGURE 5:**
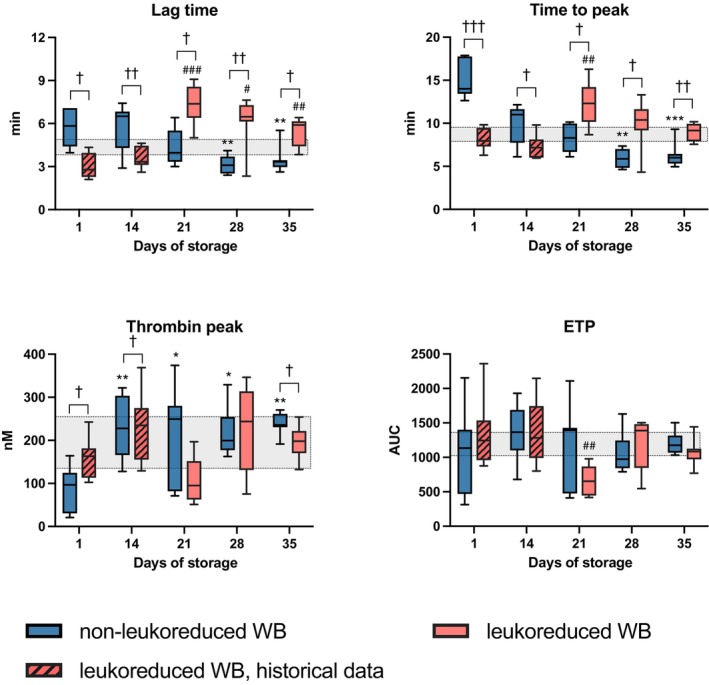
Thrombin generation in cold‐stored whole blood (CSWB). Grey area indicates baseline sample range from donors. Changes during storage in non‐leukoreduced CSWB (non‐LR‐CSWB) between day 1 and subsequent days are indicated by *p < 0.05, **p < 0.005, ***p < 0.001. Changes during storage in leukoreduced CSWB (LR‐CSWB) between day 1 and days 21, 28 and 35 are indicated by ^#^ *p* < 0.05, ^##^ *p* < 0.005, ^###^ *p* < 0.001. Differences between LR‐CSWB and non‐LR‐CSWB are indicated by ^†^ *p* < 0.05, ^††^ *p* < 0.005, ^†††^ *p* < 0.001. ETP, endogenous thrombin potential.

In non‐LR‐CSWB, lag time and time to peak shortened (*p* = 0.006 and *p* < 0.001, respectively) and thrombin peak increased (*p* = 0.008) during storage. ETP was stable (Figure 5, Figure S1).

In LR‐CSWB, lag time and time to peak prolonged until d21 before slightly shortening towards d35 (Figure 5, Figure S1). Similarly, thrombin peak and ETP were lowest on d21, markedly increasing towards d35 (*p* ≤ 0.008; Figure 5).

Comparing non‐LR‐CSWB and LR‐CSWB (Figure S1), lag time (*p* = 0.002) and time to peak (*p* = 0.004) were shorter, and thrombin peak was higher (*p* = 0.029) in non‐LR‐CSWB on d35. ETP was comparable in both groups.

### Viscoelastic tests

**FIGURE 6:**
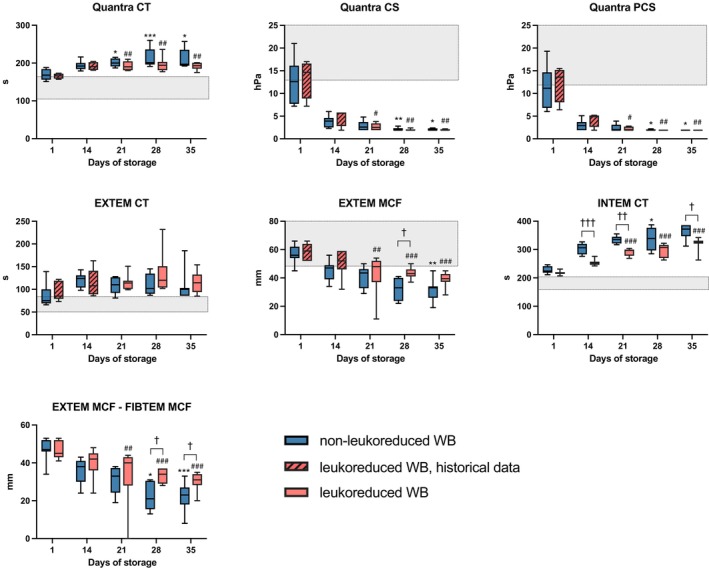
Viscoelastic assays in cold‐stored whole blood (CSWB). Grey area indicates manufacturer reference range. Quantra CS and PCS detection limit is 2.0 hPa, and results ‘<2.0 hPa’ are shown as 1.9 hPa. Changes during storage in non‐leukoreduced CSWB (non‐LR‐CSWB) between day 1 and subsequent days are indicated by *p < 0.05, **p < 0.005, ***p < 0.001. Changes during storage in leukoreduced CSWB (LR‐CSWB) between day 1 and days 21, 28 and 35 are indicated by ^#^ *p* < 0.05, ^##^ *p* < 0.005, ^###^ *p* < 0.001. Differences between LR‐CSWB and non‐LR‐CSWB are indicated by ^†^ *p* < 0.05, ^††^ *p* < 0.005, ^†††^ *p* < 0.001. CS, clot stiffness; CT, clotting time; MCF, maximum clot firmness; PCS, platelet contribution to CS.

In non‐LR‐CSWB, Quantra CT prolonged (*p* = 0.007) and CS and PCS decreased notably (*p* = 0.009) during storage (Figure 6). ROTEM EXTEM CT prolonged, but the change was not statistically significant. INTEM CT prolonged (*p* = 0.043). EXTEM MCF declined (*p* = 0.017), with abnormal median values already on d14 (Figure 6). No changes were observed in FIBTEM A5, whereas FCS in Quantra decreased slightly (*p* > 0.05; Figure 2). EXTEM‐FIBTEM MCF declined during storage (*p* = 0.003). CSL could not be measured from all samples, with increasing frequency towards the end of storage (data not shown).

In LR‐CSWB, Quantra CT prolonged in early storage but not thereafter (Figure 6). CS and PCS decreased rapidly and were immeasurably low on d28 and d21, respectively. ROTEM EXTEM CT prolonged from 85 to 115 s, but the change was not statistically significant. INTEM CT prolonged especially during early storage (Figure 6). EXTEM MCF and EXTEM‐FIBTEM MCF declined. Quantra FCS declined (*p* = 0.016), but no change in FIBTEM A5 was observed (Figure 2). CSL could not be measured from all samples, with increasing frequency towards the end of storage (data not shown).

Comparing non‐LR‐CSWB and LR‐CSWB (Figure 6), Quantra CT and EXTEM CT were similar, whereas INTEM CT was slightly longer in non‐LR‐CSWB. We observed no difference between the groups in Quantra CS, Quantra PCS or FIBTEM A5. EXTEM MCF and EXTEM‐FIBTEM MCF were higher in LR‐CSWB.

## DISCUSSION

WB used in the civilian setting is typically cold‐stored, often for 14–21 days, although there is no consensus on the time limit for LTOWB cold storage. The increased storage time from 21 days for CPD to 35 days for CPDA‐1 is mostly based on improved RBC survival and functionality due to the addition of adenine and increased glucose concentration in CPDA‐1 [19, 20]. We designed a study to better understand the haemostatic properties of CSWB during extended storage. Our results suggest that leukoreduction does not play a critical role in the haemostatic properties of CSWB and that regardless of leukoreduction, the most notable haemostatic changes occur during the first 14 storage days, with continued albeit slower decline thereafter. Platelets contribute to the clot formed even in 35‐day‐old CSWB. However, the factor most affecting the haemostatic capacity of a single CSWB unit is storage time—the fresher blood, the better.

While the platelet counts in LR‐CSWB decreased as expected [10, 12, 13, 21], we observed a rather low but stable platelet count in non‐LR‐CSWB. The paradoxically increasing platelet counts in some older non‐LR‐CSWB samples suggest that there may have been platelet aggregates in the samples drawn early during storage, undetected as we did not perform microscopy. Indeed, the platelet counts in non‐refrigerated non‐leukoreduced WB remain doubly as high [22], suggesting that cold storage induces formation of platelet aggregates [23], whereas leukoreduction may have filtered out similar aggregates in LR‐CSWB. Additionally, the analyser may have misinterpreted leukocyte fragments as platelets, thus providing an arbitrarily high platelet count towards the end of storage.

Despite the lower‐than‐expected initial platelet yield, the early aggregation response was better in non‐LR‐CSWB than in our historical LR‐CSWB samples, with significantly higher platelet counts. This may be explained by several factors: leukoreduction filtering out active and aggregated platelets in LR‐CSWB, the contribution of leukocytes to platelet aggregation in non‐LR‐CSWB and earlier cooling of non‐LR‐CSWB units. While the end‐of‐storage aggregation responses were almost non‐existent in both LR‐CSWB and non‐LR‐CSWB, this does not preclude remnant platelet activity, as the used MEA agonists detect platelet aggregation mediated only by ADP and thrombin receptor PAR‐1 (TRAPtest). There are other mechanisms resulting in platelet aggregation, including but not limited to PAR‐4 and VWF‐mediated activation [24]. The PAR‐4 pathway requires abundant thrombin, but as ETP was within control range on d35 and fibrinogen levels remained stable in both groups during storage, there may have been enough thrombin to activate platelets and fibrinogen to enable GPIIb/IIIa‐mediated platelet aggregation. Also, platelet GPIb‐IX‐V binds VWF. While the measured VWF antigen levels remain stable during WB storage, VWF‐GPIb activity is decreased, precluding any definitive conclusions on the role of VWF in this setting. Whether platelet aggregation through these mechanisms is significant in CSWB is not elucidated in our study and requires further research.

For a more complete picture of WB clot formation, we performed sonorheometry Quantra in addition to ROTEM. As the methods measuring clot strength are different, Quantra could be more sensitive to clot heterogeneity and instability [25]. While ROTEM and Quantra are not interchangeable, EXTEM MCF and Quantra CS are highly correlated [26]. However, Quantra seems more sensitive to coagulopathy (as defined by international normalized ratio (INR)) while ROTEM better detects hypofibrinogenaemia [26]. Both Quantra CS/PCS and EXTEM MCF decreased during storage, likely due to decreased platelet counts and function, as thrombin generation and fibrinogen levels are retained on d35, and FXIII, released from activated platelets and responsible for cross‐linking fibrin and stabilizing the clot, increases during storage. EXTEM‐FIBTEM MCF also decreases during storage, but at a slower rate than PCS. However, EXTEM‐FIBTEM MCF as a platelet contribution marker is ambiguous and should be interpreted with caution [27]. Indeed, CS/PCS decline to immeasurably low levels in some LR and non‐LR samples already on d21. Despite this, some platelet contribution is clearly maintained as a clot forms in ROTEM in the presence of platelets, as further affirmed by the very low EXTEM MCF if only RBCs and plasma are combined [15]. However, based on sonorheometry, clot structure may be weaker than previously thought already in 14‐day‐old WB. Further, late‐storage FIBTEM remains normal, whereas Quantra FCS decreases markedly, suggesting that cytochalasin D used in FIBTEM does not block all platelet activity and that there thus are somewhat functional platelets in ageing CSWB [28]. Clot heterogeneity may also explain the disturbed Quantra CSL towards the end of storage. Our samples were unfiltered and thus likely contain cell aggregates formed during storage, possibly causing disruptions in clot structure.

Leukocyte count in non‐LR‐CSWB decreased during storage, as expected [10, 11, 21], with 40% remaining on d35. As 50%–70% of circulating leukocytes are neutrophils viable only a few days in stored blood [29, 30], it seems likely that the reduction in leukocyte count is mostly attributable to neutrophil loss. Neutrophil extracellular traps (NETs), that is, a scaffold of DNA, histones and antimicrobial proteins released by viable or dying neutrophils [31], appear in stored RBCs [32] and are similarly likely to appear in non‐LR‐CSWB [33]. NETs act procoagulatively by activating both intrinsic and extrinsic pathways in addition to forming a scaffold promoting thrombus formation [34]. Surprisingly, late‐storage EXTEM MCF was higher in LR‐CSWB than non‐LR‐CSWB. To the best of our knowledge, there are no studies on NETs in CSWB, meriting future research.

Long cold storage affects many clotting factors [12, 13, 15]. Expectedly, FV and FVIII levels decreased, reflected in the prolonged APTT and INTEM CT. Surprisingly, these changes were more pronounced in non‐LR‐CSWB although it was refrigerated immediately in contrast to LR‐CSWB cold‐stored only after leukoreduction after 18–24 h in room temperature. This phenomenon, in line with the findings of Sivertsen et al. [12], requires future exploration. A decrease in FX levels and a concurrent decrease in PT, lower in LR‐CSWB, was also observed. Accordingly, EXTEM CT and Quantra CT were prolonged. Despite the clotting factor deficiencies implying thrombin formation may be diminished, ETP remained within control range even on d35 [10]. A possible explanation is that extracellular vesicle and NET (in non‐LR‐CSWB) formation during storage may increase available negatively charged surfaces facilitating thrombin formation. Supporting this, lag time and time to peak decreased and peak thrombin formation increased with progressing storage.

Despite some statistically significant differences, mostly in favour of LR‐CSWB, neither storage solution nor leukoreduction seem to play a significant role in the haemostatic capacity of older WB. In routine clinical use, LR‐CSWB should be used to avoid, for example, febrile non‐haemolytic transfusion reactions, HLA‐immunization and cytomegalovirus infections [2, 35]. It is probably beneficial to transfuse CSWB in routine clinical use as fresh as possible, as most haemostatic changes occur within the first 2 weeks of storage [36, 37]. However, to prepare for circumstances where blood processing and distribution are disturbed or insufficient, we second Sivertsen et al. [12]: There should be a commercial WB CPDA‐1 collection set with a built‐in leukoreduction filter. Such a set could benefit remote areas with no possibility of frequent WB donations, simultaneously reducing WB wastage. For bleeding patients in shock, the reduced clot formation capacity due to ageing platelets in up to 5‐week‐old WB does not seem a problem when the alternative is no platelets at all [37].

There are several limitations to our study. First, the sample size was small. This accurately reflects the individual variance between donors and, thus, WB units and the achieved transfusion response. Second, we recruited donors only for non‐LR‐CSWB group and utilized expired surplus LTOWB for LR‐CSWB group, as recruiting more donors for research purposes only would keep them from donating for clinical needs, which is the primary responsibility of FRCBS. LTOWB was available for samples from d21 onwards. We therefore used data from previous studies for d1 and d14 in the LR‐CSWB group [15, 16]. This may impact the accuracy of statistical comparison. Third, we cannot deduce whether the observed differences are caused by storage medium (CPD vs. CDPA‐1) or leukoreduction, or the combination of both. However, storage medium seems to have only minimal effects on in vitro haemostasis [10, 13]. Fourth, due to the experimental nature of the study, we cannot draw conclusions about the clinical significance of our results. Platelet function in the end‐of‐storage CSWB appears impaired, and in vivo studies should be conducted to elucidate whether the loss of function is at least partly reversible [38].

In conclusion, the most detrimental haemostatic changes in LR‐CSWB and non‐LR‐CSWB occur during the first 14 days of storage. Clot structure may be weaker than previously thought already in 14‐day‐old WB. These findings imply that, to achieve the best haemostatic effect, WB should be used as fresh as possible. However, our results suggest that even 35‐day‐old WB is better than the transfusion of RBCs and plasma without platelets. We await clinical studies on the haemostatic effects of WB and how the age of the transfused WB affects the procoagulative state of the patient.

## CONFLICT OF INTEREST STATEMENT

T.H. received honoraria from Werfen and Swedish Orphan Biovitrum.

## Supporting information


**Figure S1.** Calibrated automated thrombogram curves in non‐leukoreduced and leukoreduced cold‐stored whole blood.

## Data Availability

The data that support the findings of this study are available from the corresponding author upon reasonable request.
