# Supplementary material for: The effect of leukoreduction and prolonged storage on coagulation in cold‐stored whole blood: An in vitro study
Source: Vox Sang. 2025 Aug 5;120(9):881–91. doi: 10.1111/vox.70075 (PMC12422834; doi:10.1111/vox.70075)

Supplementary Figure S1. Calibrated automated thrombogram curves in non-leukoreduced and leukoreduced cold-stored whole blood.

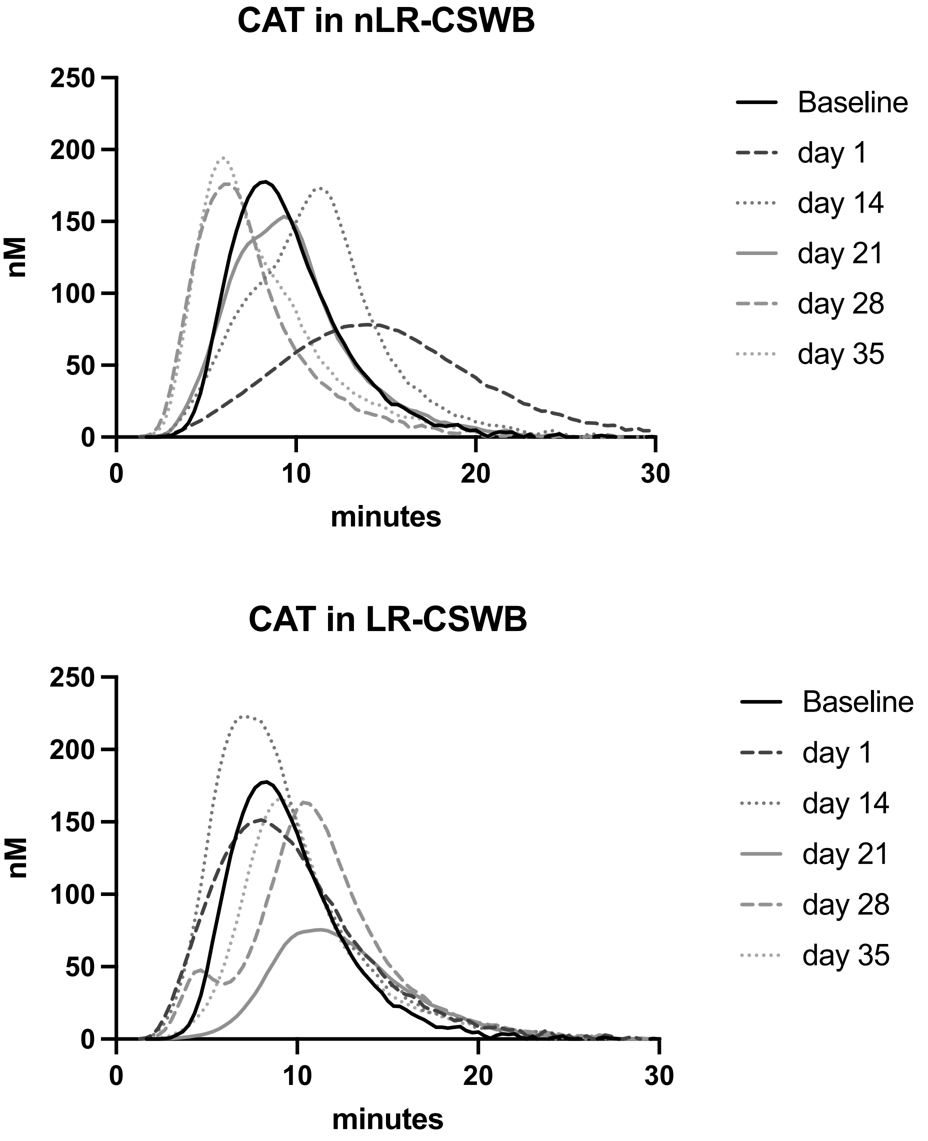

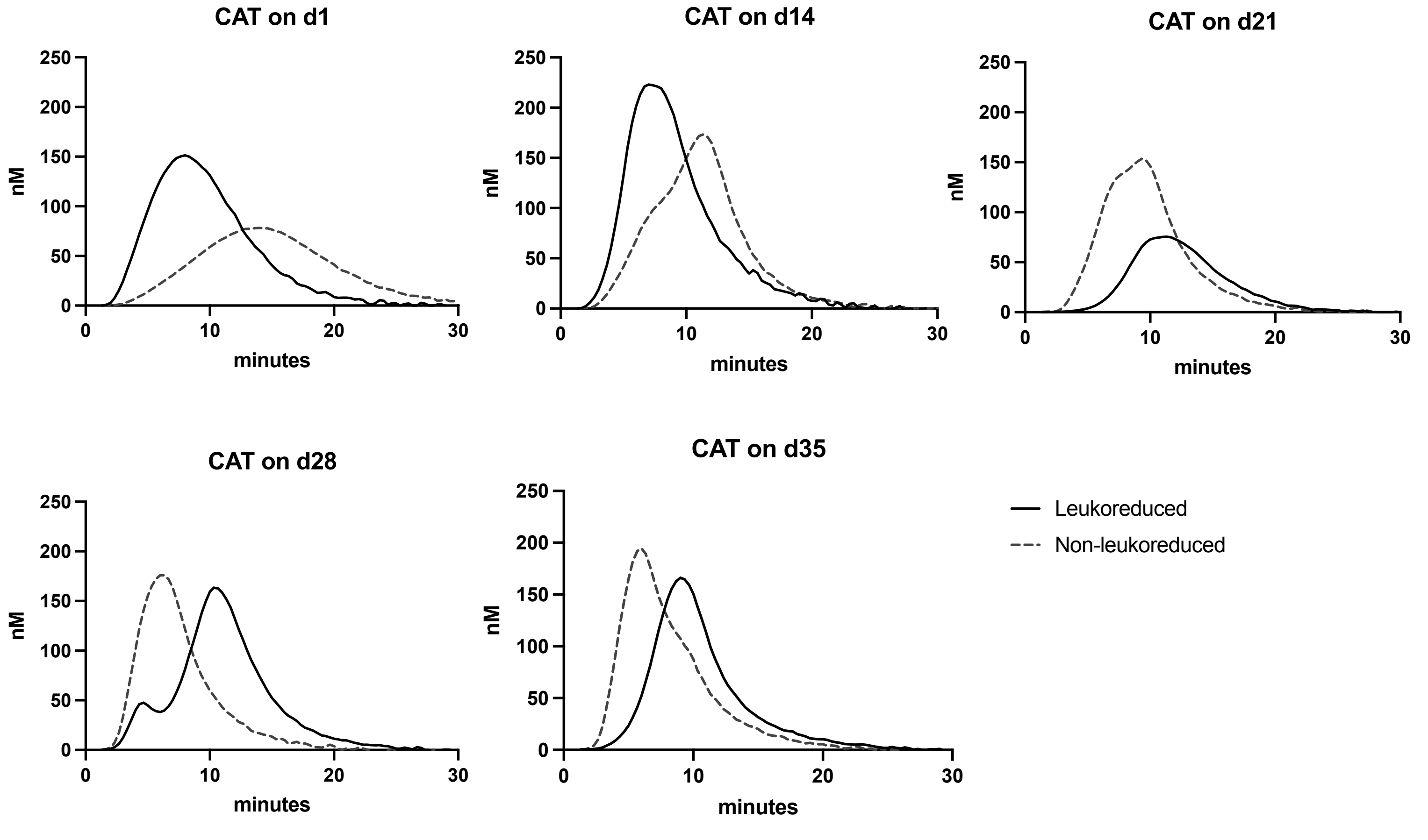

Supplement: Supplementary file 1 — Figure S1. Calibrated automated thrombogram curves in non‐leukoreduced and leukoreduced cold‐stored whole blood. [file VOX-120-881-s001.pdf]
